# Efficacy of PD-1/PD-L1 inhibitors in gastric or gastro-oesophageal junction cancer based on clinical characteristics: a meta-analysis

**DOI:** 10.1186/s12885-023-10605-y

**Published:** 2023-02-10

**Authors:** Gengwei Huo, Wenjie Liu, Peng Chen

**Affiliations:** 1 Department of Thoracic Oncology, Tianjin Medical University Cancer Institute and Hospital, National Clinical Research Center for Cancer, Key Laboratory of Cancer Prevention and Therapy of Tianjin, Tianjin’s Clinical Research Center for Cancer, Tianjin, 300060 China; 2 Department of Oncology, Jining NO.1 People’s Hospital, Jining, 272000 Shandong China

**Keywords:** Immune checkpoint inhibitor, Gastroesophageal junction cancer, Gastric cancer, Efficacy, Predictor, Meta-analysis

## Abstract

**Purpose:**

Programmed death-1 (PD-1) and its ligand (PD-L1) inhibitors have been reported in several clinical trials for gastric cancer and gastroesophageal junction cancer (GC/GEJC). We presently carried out a meta analysis to evaluate the potency of PD-1/PD-L1 inhibitors in advanced GC/GEJC individuals with different clinical features and to determine patients more probably benefiting from the treatment.

**Methods:**

Randomized clinical trials (RCTs) in databases that compared PD-1/PD-L1 inhibitors to chemotherapy in patients with GC/GEJC published before May 2022 were retrieved. Basic characteristics were extracted from the included studies as well as hazard ratios (HR) and 95 percent confidence intervals (CI) for all individuals and subgroups. The inverse variance weighting method was used to evaluate pooled treatment data.

**Findings:**

Four RCTs involving 2,253 individuals were included. The results suggested that PD-1/PD-L1 inhibitors substantially enhanced overall survival (OS) (HR, 0.91; CI 95%, 0.83–1.00; *p* = 0.04) but not progression free survival (PFS) (HR, 1.17; CI 95%, 0.83–1.64; *p* = 0.38) in GC/GEJC individuals compared with chemotherapy. Significantly improved OS was observed in individuals aged < 65 years (HR, 0.84; *p* = 0.003), and men (HR, 0.88; *p* = 0.02), but not in individuals aged ≥ 65 years (HR, 0.97; *p* = 0.62), and women (HR, 0.98; *p* = 0.82).

**Implications:**

PD-1/PD-L1 inhibitors improve OS but not PFS compared with chemotherapy in GC/GEJC. Age and sex could be used to predict the treatment potency of PD-1/PD-L1 inhibitors in GC/GEJC.

**Supplementary Information:**

The online version contains supplementary material available at 10.1186/s12885-023-10605-y.

## Introduction

Gastric or gastroesophageal junction cancer (GC/GEJC) is the third common cause of cancer-related deaths globally and the fifth most prevalent, with 63% of these cases being locally advanced or metastatic, and more than one million cases were newly diagnosed in 2018 [1, 2]. Currently, patients with GC are mainly treated with surgery and chemotherapy [3–7]. However, most patients with advanced GC/GEJC continue to progress after receiving treatment and have a poor prognosis [8]. The median overall survival (OS) of individuals with HER2-negative GC is 8–12 months [9], while the median OS of individuals with HER2-positive GC is as high as 13 months. These individuals may benefit from 1st-line targeted therapy [10], where treatment choices were further constrained [3, 11–13]. Subsequently, immunotherapy has rapidly developed and gradually become a new frontier of advanced GC/GEJC therapy due to the genetic complexity and heterogeneity of cancer, wholly innovating the treatment outlook over the past decade, in which immune escape has played a significant role [14]. Immune checkpoint inhibitors (ICIs), also known as programmed death-1 (PD-1) or its ligand (PD-L1), as promising cancer therapeutic targets in the field of oncology, have caused great concern [15]. ICIs target critical regulators that help tumor cells escape immune attack, thereby enhancing the cytotoxic activity of anti-tumor T cells [16, 17]. To date, several extensive randomized controlled trials (RCTs) have confirmed that many tumor types can achieve disease control and improve OS from PD-1/PD-L1 inhibitors treatment [18–21]. This rekindled our interest in targeting GC/GEJC patients with ICIs.

However, the response obtained from ICIs is observed in only a minority of GC/GEJC patients (approximately 10 to 20%) [22], and there may be potential for drug resistance and rapid progression of the disease. In addition, existing findings from different clinical trials in this area appear to be controversial, and there is no clear conclusion regarding the role of immunotherapy in individuals with GC/GEJC. Therefore, it is becoming even more urgent to look for dependable predictors to help clinicians screen individuals who are more likely to benefit from ICIs, both to restrict the number of patients exposed to latent autoimmunity by the effects of targeted axis drugs and to protect patients from ineffective treatment [23].

The expression of PD-L1 has been seen as a prognostic biomarker, which is expressed in 30 to 65% of invasive GCs, and is associated with some factors, such as EBV infection, tumor size, tumor depth of invasion, lymph nodes, and distant metastasis [24–26]. Currently, PD-L1 positive expression as a biomarker for pembrolizumab 3rd-line treatment regimen in GC has been approved by the FDA [27], and nivolumab has been approved in many regions as a treatment regimen for unresectable late or recurrent GC, regardless of PD-L1 expression, which indicates that the potential and application range of immunotherapy are expanding. Unfortunately, a predictive biomarker for anti-PD-1/PD-L1 antibodies therapy, PD-L1 level, remains challenging in clinical trials. For instance, PD-L1 in GC/GEJC was defined according to the combined positive score (CPS), involving the expression of tumor cells, macrophages, and lymphocytes, and this differs from the PD-L1 definition in carcinoma of the lungs [28]; The cut-off value of PD-L1 positive expression has not been determined; PD-L1 expression is impacted by a number of factors, such as anti-tumor therapy, standardized detection methods, and the immune response of the host [29, 30]. Another controversial biomarker is tumor mutation burden (TMB). Studies have shown that different TMB levels in GC lead to different therapeutic effects and prognoses [31, 32]. Therefore, whether TMB has an ideal truncation value and its predictive effect on GC/GEJC requires further study.

It makes sense to look for other practical and economic biomarkers to predict the potency of PD-1/PD-L1 inhibitors in GC/GEJC. In previously published meta-analyses, the potency of immunotherapy against PD-1/PD-L1 was found to be superior to that of chemotherapy in the treatment of advanced gastric esophageal cancer [33–35]. These conclusions were drawn after the inclusion of patients with esophageal cancer (EC) who received ICIs or chemotherapy; thus, immunotherapy has a generally good prognosis. However, accurate predictions of the GC/GEJC should not be obtained from such comparisons. Several studies have shown that immunotherapy has a remarkable effect on EC [36, 37], which may lead to false-positive results when included in analyses based on GC. Therefore, we performed a meta-analysis that only included individuals with GC/GEJC accepting PD-1/PD-L1 inhibitors, and all those individuals were compared. The CheckMate 649 trial [38] that involved patients with EC was not included. We could scientifically determine which factors among the different clinical characteristics make GC/GEJC individuals more likely to benefit from anti-PD-1/anti-PD-L1 antibodies and guide the choice of treatment. We present these results based on the Preferred Reporting Items for Systemic Reviews and Meta-Analyses (PRISMA) guidelines [39].

## Methods

### Inclusion and exclusion criteria

Articles that met the inclusion and exclusion criteria were selected in accordance with the PICOs structure (participants, intervention, comparison, and outcomes). We screened articles by carefully examining their titles, abstracts, and full texts. Duplicate articles and irrelevant studies were excluded. Inclusion criteria that need to meet: (I) RCTs to explore the potency of (nivolumab, pembrolizumab, toripalimab, remelimumab, relatlimab, cemiplimab, avelumab, durvalumab, atezolizumab, PD-1, PD-L1, immunotherapy, or immune checkpoint inhibitors or ICIs) compared with chemotherapy for individuals with (gastric cancer, stomach cancer, gastroesophageal junction cancer, gastroesophageal junction cancer, GC, GEJC, esophagogastric junction cancer, or esophagogastric junction cancer), (II) hazard ratio (HR) and corresponding 95 percent confidence interval (95% CI) to OS and/or PFS of some pre-defined subpopulations were reported; (III) if more than one article reported the same RCTs, adopting the most recent data, the longest follow-up time, and the maximum number of patients population; (IV) if some of the studies came from the same RCTs, we included all the different subgroups.

Studies were excluded if they met any of the following criteria: (I) following publication types: case reports, letter, study protocol, animal and cellular studies, editorials or reviews, (II) inadequate existing survival data, or without dose and usage information; (III) except anti-PD- (L) 1 or chemotherapy, the patients were treated with placebo, radiotherapy, vaccine therapy, and other therapies.

### Literature searching and data collecting

Study selection and data extraction were performed independently by the two authors (WJL and GWH) and then checked with each other. If any ambiguity was encountered, a third author should be sought (PC). Systematic filtering of potentially relevant publications was conducted using PubMed, EMBASE, Cochrane Library, Medline, and major oncology conferences. Complied with the search criteria, we searched articles from their inception to May 2022. Information was recorded for each study as follows: publication year, name of first author, trial name, study phase, line of therapy, clinical trial number, usage and dosage, age composition, gender composition, Eastern Cooperative Oncology Group (ECOG) performance status (PS) score, PD-L1 expression level, trial design, blinding, and primary endpoint, together with survival outcome measurements of pre-defined subgroups.

### Quality assessment and statistical analyses

GWH and WJL independently evaluated the reliability and validity of RCTs using the Cochrane bias tool. Review Manager 5.3 was applied for our overall statistical analysis. The main endpoint was the OS of PD-1/PD-L1 inhibitors compared with that of non-ICI therapy. The secondary endpoint was PFS. According to RCTs heterogeneity, a fixed-effect model or random-effect model was used to obtain HR. When heterogeneity was acceptable (*I*^*2*^ < 50% and *p* > 0.10), we adopted the model of fixed effect, otherwise we adopted the model of random effect. The *I*^*2*^ statistics test and chi-square test were used as existing heterogeneity indicators. The results were presented in the form of forest plots, with aggregate assessments and a 95 percent CI corresponding to those assessments. If the study had a small sample size, it was excluded from sensitivity analysis. Statistical significance was set at *p* < 0.05, and the *p* values were bilateral.

## Results

### Study selection and features

**Fig. 1:**
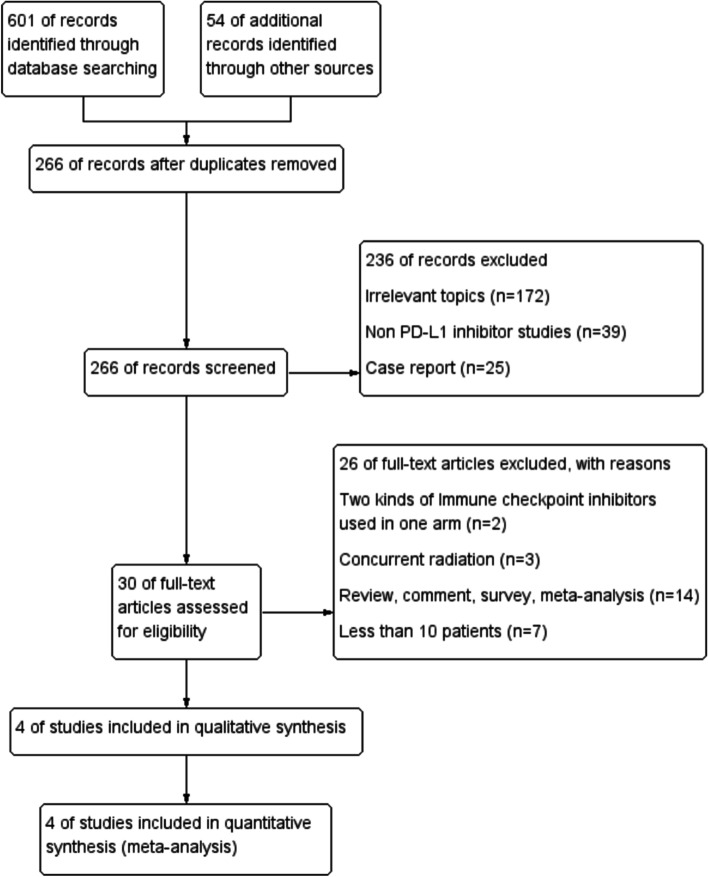
PRISMA flow diagram

**Fig. 2:**
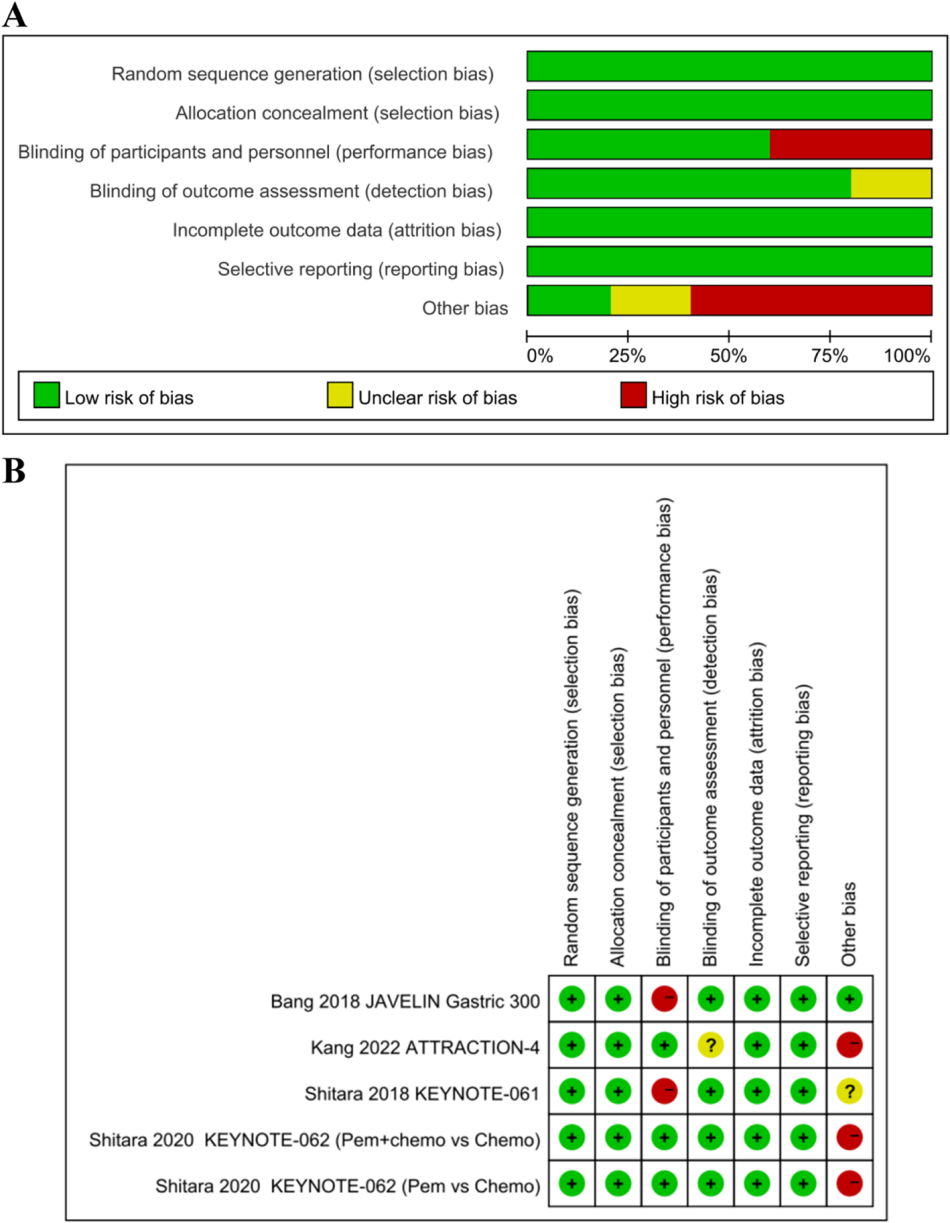
Assessment of bias risk, (**A**) risk of bias graph, (**B**) risk of bias summary

The retrieval strategy applied in this study identified 655 potentially relevant records from databases and conferences. Figure 1 describes the process of selection and the reasons for removing studies considered unacceptable. After screening the abstracts and full texts, 651 studies were excluded. Therefore, four phase III articles [40–43] involving 2,253 individuals with GC/GEJC were included, and these RCTs were published between 2018 and 2022. The KEYNOTE-062 study included two assessable experimental anti-PD-1-based treatment arms, which were all compared with the control group chemotherapy. It should be noted that we separately used two trials group to describe the results of the KEYNOTE-062 experiment in the following content. Three trials were conducted in the 1st-line (KEYNOTE-062 arm A; KEYNOTE-062 arm B; ATTRACTION-4), one in the 2nd-line (KEYNOTE-061), and one in the 3rd-line (JAVELIN Gastric 300). Table 1 presents the basic features of the studies included. The analysis of each subgroup indicated little heterogeneity. Explicit bias risk analysis showed that the bias risk for all RCTs was low (Fig. 2).

**Table 1:** Basic characteristics of included studies

**Reference**	**Trial**	**Study phase**	**Stage**	**Treatment line**	**PD-1/PD-L1 (n)**	**Control (n)**	**Primary endpoint**	**Median (range)** **Age (years)**		**Males (%)**		**Tumor site**				**ECOG**			
								**PD-1/ PD-L1**	**Control**	**PD-1/ PD-L1**	**Control**	**GC (%)**		**GEJC (%)**		**PD-1/ PD-L1**		**Control**	
												**PD-1/ PD-L1**	**Control**	**PD-1/ PD-L1**	**Control**	**0 (%)**	**1 (%)**	**0 (%)**	**1 (%)**
Bang et al. 2018 [40]	JAVELIN Gastric 300	III	Advanced	3L	Avelumab (185)	Physician’s choice of chemotherapy (186)	OS	59 (29–86)	61 (18–82)	75.7	68.3	65.9	74.2	34.1	25.8	35.7	64.3	33.3	66.7
Shitara et al. 2018 [41]	KEYNOTE-061	III	Advanced	≥ 2L	Pembrolizumab (196)	Chemotherapy (199)	OS and PFS	64 (57–70)	61 (54–68)	74	70	68	63	32	37	45	55	46	53
Shitara et al. 2020 Arm A [42]	KEYNOTE-062	III	Advanced	1L	Pembrolizumab (256)	Chemotherapy (250)	OS and PFS	61.0 (20–83)	62.5 (23–87)	70.3	71.6	68.8	72.4	30.9	26.8	51.2	48.8	46.0	54.0
Shitara et al. 2020 Arm B [42]					Pembrolizumab + Chemotherapy (257)			62.0 (22–83)	62.5 (23–87)	75.9	71.6	66.1	72.4	33.1	26.8	46.3	53.7	46.0	54.0
Kang et al. 2022 [43]	ATTRACTION-4	III	Advanced or recurrent	1L	Nivolumab + Chemotherapy (362)	Placebo + Chemotherapy (362)	PFS and OS	64 (25–86)	65 (27–89)	70	75	65	66	8	9	54	46	54	46

### Effects of PD-1/PD-L1 Inhibitors for GC/GEJC

**Fig. 3:**
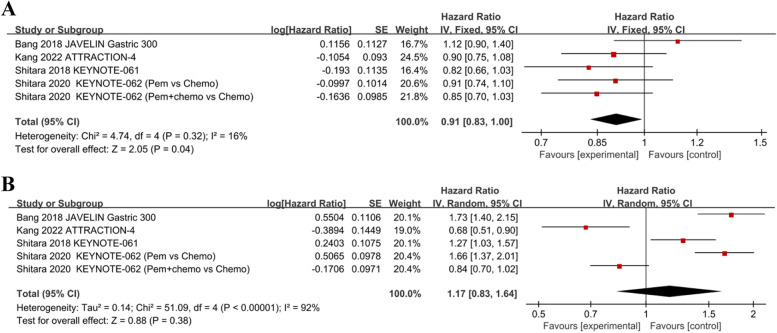
Forest plots of HRs comparing (**A**) OS and (**B**) PFS between anti-PD-1/PD-L1 therapy and chemotherapy

**Fig. 4:**
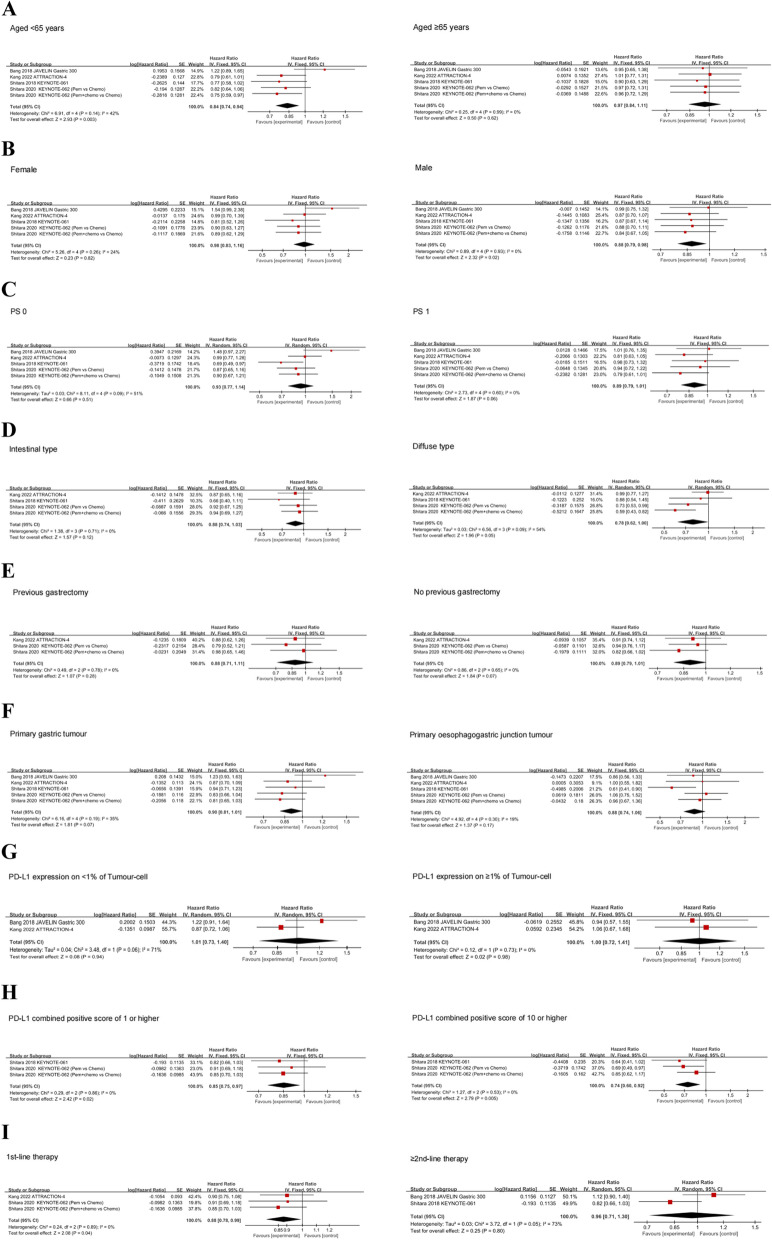
Forest plots of HRs comparing OS between anti-PD-1/PD-L1 therapy and chemotherapy with respect to (**A**) age group, (**B**) gender, (**C**) PS score, (**D**) Lauren histological type, (**E**) previous gastrectomy status, (**F**) primary tumour sites, (**G**) PD-L1 TPS, (H) PD-L1 CPS, (**I**) treatment line

Five trials examined the potency of immunotherapy in patients with GC/GEJC. The comprehensive findings suggested that PD-1/PD-L1 inhibitors substantially enhanced OS (HR, 0.91; 95% CI, 0.83–1.00; *p* = 0.04) (Fig. 3A). However, ICIs did not achieve improve in PFS compared to chemotherapy in these individuals (HR, 1.17; 95% CI, 0.83–1.64; *p* = 0.38) (Fig. 3B). Pooling the results from three trials, patients receiving 1st-line treatment with PD-1/PD-L1 inhibitors had improved OS (HR, 0.88; CI 95%, 0.78–0.99; *p* = 0.04), but no substantial OS improvement was found in individuals who received ≥ 2nd-line therapy (HR, 0.96; CI 95%, 0.71–1.30; *p* = 0.80) compared to chemotherapy (Fig. 4I and Table 2).

**Table 2:** Analyses of OS in subgroups of patients with different clinical features

Population	Subgroup	No. of studies	Test of association			Test of heterogeneity	
			HR	95% CI	*p* value	I^2^	*p* value
Aged < 65 years	Total	5	0.84	0.74–0.94	0.003	42%	0.14
	1st Line	3	0.79	0.68–0.91	0.001	0%	0.89
	≥ 2nd Line	2	0.96	0.61–1.51	0.87	78%	0.03
	monotherapy	3	0.91	0.70–1.18	0.48	63%	0.07
	combination therapy	2	0.77	0.65–0.92	0.004	0%	0.81
Aged ≥ 65 years	Total	5	0.97	0.84–1.11	0.62	0%	0.99
	1st Line	3	0.98	0.83–1.16	0.83	0%	0.97
	≥ 2nd Line	2	0.92	0.71–1.20	0.54	0%	0.85
	monotherapy	3	0.94	0.78–1.15	0.56	0%	0.95
	combination therapy	2	0.99	0.81–1.20	0.90	0%	0.83
Female	Total	5	0.98	0.83–1.16	0.82	24%	0.26
	1st Line	3	0.93	0.76–1.14	0.46	0%	0.91
	≥ 2nd Line	2	1.12	0.60–2.09	0.73	75%	0.04
	monotherapy	3	1.03	0.71–1.49	0.88	60%	0.08
	combination therapy	2	0.94	0.73–1.21	0.64	0%	0.70
Male	Total	5	0.88	0.79–0.98	0.02	0%	0.93
	1st Line	3	0.86	0.76–0.98	0.02	0%	0.95
	≥ 2nd Line	2	0.93	0.76–1.13	0.45	0%	0.52
	monotherapy	3	0.91	0.78–1.05	0.20	0%	0.77
	combination therapy	2	0.85	0.73–1.00	0.04	0%	0.84
PS 0	Total	5	0.93	0.77–1.14	0.51	51%	0.09
	1st Line	3	0.93	0.79–1.09	0.35	0%	0.77
	≥ 2nd Line	2	1.00	0.47–2.12	1.00	87%	0.006
	monotherapy	3	0.94	0.63–1.40	0.77	74%	0.02
	combination therapy	2	0.95	0.79–1.15	0.62	0%	0.62
PS 1	Total	5	0.89	0.79–1.01	0.06	0%	0.60
	1st Line	3	0.84	0.73–0.98	0.02	0%	0.61
	≥ 2nd Line	2	1.00	0.81–1.23	0.98	0%	0.88
	monotherapy	3	0.97	0.83–1.15	0.75	0%	0.93
	combination therapy	2	0.80	0.67–0.96	0.01	0%	0.86
Intestinal type	Total	4	0.88	0.74–1.03	0.12	0%	0.71
	1st Line	3	0.90	0.76–1.08	0.26	0%	0.94
	≥ 2nd Line	1	0.66	0.40–1.11	0.12		
	monotherapy	2	0.84	0.64–1.10	0.20	9%	0.29
	combination therapy	2	0.90	0.73–1.11	0.32	0%	0.73
Diffuse type	Total	4	0.78	0.62–1.00	0.05	54%	0.09
	1st Line	3	0.76	0.56–1.03	0.08	69%	0.04
	≥ 2nd Line	1	0.88	0.54–1.45	0.63		
	monotherapy	2	0.77	0.59–1.00	0.05	0%	0.51
	combination therapy	2	0.77	0.47–1.28	0.32	83%	0.01
Previous gastrectomy	Total	3	0.88	0.71–1.11	0.28	0%	0.78
	1st Line	3	0.88	0.71–1.11	0.28	0%	0.78
	monotherapy	1	0.79	0.52–1.21	0.28		
	combination therapy	2	0.92	0.71–1.20	0.56	0%	0.71
No previous gastrectomy	Total	3	0.89	0.79–1.01	0.07	0%	0.65
	1st Line	3	0.89	0.79–1.01	0.07	0%	0.65
	monotherapy	1	0.94	0.76–1.17	0.59		
	combination therapy	2	0.87	0.75–1.01	0.06	0%	0.50
Primary gastric tumor	Total	5	0.90	0.81–1.01	0.07	35%	0.19
	1st Line	3	0.84	0.74–0.96	0.009	0%	0.90
	≥ 2nd Line	2	1.07	0.88–1.30	0.50	47%	0.17
	monotherapy	3	0.97	0.78–1.22	0.82	57%	0.10
	combination therapy	2	0.84	0.72–0.99	0.04	0%	0.67
Primary oesophagogastric junction tumor	Total	5	0.88	0.74–1.06	0.17	19%	0.30
	1st Line	3	1.01	0.80–1.27	0.95	0%	0.92
	≥ 2nd Line	2	0.71	0.53–0.95	0.02	28%	0.24
	monotherapy	3	0.83	0.59–1.16	0.27	54%	0.11
	combination therapy	2	0.97	0.71–1.31	0.84	0%	0.90
TC < 1%	Total	2	1.01	0.73–1.40	0.94	71%	0.06
	1st Line	1	0.87	0.72–1.06	0.17		
	≥ 2nd Line	1	1.22	0.91–1.64	0.18		
	monotherapy	1	1.22	0.91–1.64	0.18		
	combination therapy	1	0.87	0.72–1.06	0.17		
TC ≥ 1%	Total	2	1.00	0.72–1.41	0.98	0%	0.73
	1st Line	1	1.06	0.67–1.68	0.80		
	≥ 2nd Line	1	0.94	0.57–1.55	0.81		
	monotherapy	1	0.94	0.57–1.55	0.81		
	combination therapy	1	1.06	0.67–1.68	0.80		
CPS ≥ 1	Total	3	0.85	0.75–0.97	0.02	0%	0.86
	1st Line	2	0.87	0.74–1.02	0.08	0%	0.70
	≥ 2nd Line	1	0.82	0.66–1.03	0.09		
	monotherapy	2	0.86	0.72–1.02	0.08	0%	0.59
	combination therapy	1	0.85	0.70–1.03	0.10		
CPS ≥ 10	Total	3	0.74	0.60–0.92	0.005	0%	0.53
	1st Line	2	0.77	0.61–0.97	0.03	0%	0.37
	≥ 2nd Line	1	0.64	0.41–1.02	0.06		
	monotherapy	2	0.67	0.51–0.89	0.005	0%	0.81
	combination therapy	1	0.85	0.62–1.17	0.32		
1st line therapy	Total	3	0.88	0.78–0.99	0.04	0%	0.89
	monotherapy	1	0.91	0.69–1.18	0.47		
	combination therapy	2	0.88	0.77–1.00	0.05	0%	0.67
≥ 2nd line therapy	Total	2	0.96	0.71–1.30	0.80	73%	0.05
	monotherapy	2	0.96	0.71–1.30	0.80		

### Age based effects

Age-specific survival data for patients with GC/GEJC were mentioned from five trials. In individuals with aged < 65 years old (HR, 0.84; CI 95%, 0.74–0.94; *p* = 0.003), compared to chemotherapy, PD-1/PD-L1 inhibitors significantly prolonged OS, but there was no survival benefit for those aged ≥ 65 years old (HR, 0.97; 95% CI, 0.84–1.11; *p* = 0.62) (Fig. 4A). Subgroup analysis based on treatment lines revealed that compared to those receiving non-ICI treatment, individuals with aged < 65 years accepting anti-PD-1/PD-L1 antibody as 1st-line therapy had better OS (HR, 0.79; 95% CI, 0.68–0.91; *p* = 0.001), but no substantial survival difference was observed in ≥ 2nd-line therapy (HR, 0.96; 95% CI, 0.61–1.51; *p* = 0.87). Subgroup analysis based on the regimen of treatment showed that there was survival benefit in individuals with aged < 65 years taking combination immunotherapy (HR, 0.77; 95% CI, 0.65–0.92; *p* = 0.004), but not in individuals receiving ICI monotherapy (HR, 0.91; 95% CI, 0.70–1.18; *p* = 0.48). Nevertheless, regardless of the treatment regimen or line of therapy, PD-1/PD-L1 inhibitors did not improve survival in individuals aged ≥ 65 years (Table 2).

### Gender based effects

Five trials tested the potency of anti-PD-1/PD-L1 antibodies in female individuals with GC/GEJC. The combined results indicated that ICI did not lead to longer OS compared to non-immunotherapy (HR, 0.98; 95% CI, 0.83–1.16; *p* = 0.82) (Fig. 4B). Subgroup analysis showed that neither the treatment line nor regimen substantially prolonged survival in these patients (Table 2). Five trials reported survival data for male, and the pooled results revealed that compared with chemotherapy, the treatment with anti-PD-1/PD-L1 antibodies markedly enhanced OS (HR, 0.88; CI 95%, 0.79–0.98; *p* = 0.02) (Fig. 4B). Subgroup analyses revealed that the integrated HR, was 0.86 (CI 95%, 0.76–0.98; *p* = 0.02) in individuals who received 1st-line treatment and 0.93 (CI 95%, 0.76–1.13; *p* = 0.45) in individuals who received ≥ 2nd-line therapy. The analysis of subgroup indicated that the integrated HR, was 0.91 (95% CI, 0.78–1.05; *p* = 0.20) with individuals who received ICI monotherapy, and 0.85 (95% CI, 0.73–1.00; *p* = 0.04) in individuals receiving combination immunotherapy by the treatment regimen (Table 2).

### ECOG PS based effects

Five trials examined the effectiveness of anti-PD-1/PD-L1 antibody therapy for GC/GEJC patients with PS 0 and PS 1. Our integrated findings revealed that compared to chemotherapy, patients with both PS 0 (HR, 0.93; CI 95%, 0.77–1.14; *p* = 0.51) and PS 1 (HR, 0.89; CI 95%, 0.79–1.01; *p* = 0.06) failed to realize OS enhancements after applying the treatment with PD-1/PD-L1inhibitors (Fig. 4C). Unexpectedly, the analyses of subgroup shown that in patients with PS 1, receiving 1st-line therapy (HR, 0.84; 95% CI, 0.73–0.98; *p* = 0.02) and ICI-based combination therapy (HR, 0.80; 95% CI, 0.67–0.96; *p* = 0.01) significantly prolonged OS compared with non-ICI, but not in monotherapy or ≥ 2nd-line treatment. Subgroup analyses revealed that neither the regimen of therapy nor the treatment line could prolong the survival of individuals with PS 0 (Table 2).

### Histological type based effects

PD-1/PD-L1 inhibitors were not observed to improve OS better than chemotherapy, either in intestinal type (HR, 0.88; 95% CI, 0.74–1.03; *p* = 0.12) or diffuse type (HR, 0.78; 95% CI, 0.62–1.00; *p* = 0.05) GC/GEJC patients (Fig. 4D). The subgroup analyses showed that survival improvement was not observed in individuals with either the intestinal or diffuse type, regardless of the regimen or line of treatment (Table 2).

### Gastrectomy status based effects

Three trials provided survival data in GC/GEJC individuals with different gastrectomy states; both studies examined the potency of PD-1/PD-L1 inhibitors as 1st-line treatment. The pooled HRs of individuals with a history of gastrectomy and no history of gastrectomy were 0.88 (95% CI, 0.71–1.11; *p* = 0.28) and 0.89 (95% CI, 0.79–1.01; *p* = 0.07), respectively (Fig. 4E). Analysis of subgroups according to the treatment regimen showed that individuals with and without gastrectomy, accepting anti-PD-1/PD-L1 therapy, and those accepting non-ICI had no substantial statistical difference in survival (Table 2).

### Primary tumor sites based effects

In individuals with primary GC/GEJC, OS outcomes were reported in five trials. The integrated findings revealed that PD-1/PD-L1 inhibitors did not apparently prolong OS (HR, 0.90; CI 95%, 0.81–1.01; *p* = 0.07) or GEJC (HR, 0.88; CI 95%, 0.74–1.06; *p* = 0.17) (Fig. 4F). However, subgroup analysis revealed that for individuals with GC, the pooled HR, was 0.84 (95% CI, 0.74–0.96; *p* = 0.009) for those who received 1st-line treatment, and 0.84 (95% CI, 0.72–0.99; *p* = 0.04) for those who received combination therapy, which markedly prolonged OS compared with non-ICI. For patients with GEJC, analyses of subgroups by the line of treatment revealed that anti-PD-1/PD-L1 antibody only benefits from the treatment of ≥ 2nd-line (HR, 0.71; 95% CI, 0.53–0.95; *p* = 0.02), while those who received 1st-line did not. Neither monotherapy nor combination therapy benefited patients with GEJC, according to the treatment regimen (Table 2).

### Tumor Cell PD-L1 expression based effects

The potency of PD-1/PD-L1 inhibitors for GC/GEJC patients with the expression of PD-L1 on tumor cells (TC) < 1% or ≥ 1% was studied in two researches, respectively. The pooled results revealed that immunotherapy did not markedly enhance OS in individuals with TC < 1% (HR, 1.01; CI 95%, 0.73–1.40; *p* = 0.94) or TC ≥ 1% (HR, 1.00; CI 95%, 0.72–1.41; *p* = 0.98) (Fig. 4G). Subgroup analyses showed that, regardless of the line or regimen of the treatment, the survival did not prolong both in individuals TC < 1% and ≥ 1% compared to chemotherapy (Table 2).

### PD-L1 CPS based effects

There were three trials that looked at the potency of PD-1/PD-L1 inhibitors in GC/GEJC individuals with PD-L1 CPS (combined positive score) ≥ 1, and the integrated results revealed that there was substantial difference in OS between immunotherapy and non-ICI therapy (HR, 0.85; CI 95%, 0.75–0.97; *p* = 0.02) (Fig. 4H). As for individuals with PD-L1 CPS ≥ 10, the effectiveness of PD-1/PD-L1 inhibitors has been demonstrated to be the same in three trials. OS was substantially improved by PD-1/PD-L1 inhibitors compared with chemotherapy (HR, 0.74; CI 95%, 0.60–0.92; *p* = 0.005), according to the pooled data (Fig. 4H). The analysis of subgroup revealed that the integrated HR, was 0.77 (95% CI, 0.61–0.97; *p* = 0.03) in individuals accepting 1st-line therapy, but 0.64 (95% CI, 0.41–1.02; *p* = 0.06) in individuals who received ≥ 2nd-line treatment in individuals with PD-L1 CPS ≥ 10 by the treatment line. The analysis of subgroup revealed that in those patients, compared with chemotherapy, anti-PD-1/anti-PD-L1 monotherapy substantially prolonged survival (HR, 0.67; 95% CI, 0.51–0.89; *p* = 0.005), while no OS improvement was observed for individuals receiving pembrolizumab combined with chemotherapy only from one study (HR, 0.85; 95% CI, 0.62–1.17; *p* = 0.32) by the regimen of treatment (Table 2).

### Drug selection

Table 3 illustrates how GC/GEJC patients with certain characteristics could choose a more appropriate treatment regimen as well as the more likely range of action of the anti-PD-1/PD-L1 antibody itself. Based on our subgroup results, PD-1/PD-L1 inhibitors combined with chemotherapy as 1st-line treatment is recommended for patients aged < 65 years, male, PS 1, and primary GCs. ICI 1st-line therapy or monotherapy is recommended for individuals with a PD-L1 CPS of ≥ 10. Primary GEJCs might be more efficient when PD-1/PD-L1 inhibitors as a ≥ 2st-line treatment.

**Table 3:** Different treatment regimens with OS benefited from ICI-based therapy over chemotherapy in targeted patients

Regimen	Population	No. of studies	Test of association			Test of heterogeneity	
			HR	95% CI	*P* value	I^2^	*P* value
1st-Line	Aged < 65 years	3	0.79	0.68–0.91	0.001	0%	0.89
	Male	3	0.86	0.76–0.98	0.02	0%	0.95
	PS 1	3	0.84	0.73–0.98	0.02	0%	0.61
	gastric tumor	3	0.84	0.74–0.96	0.009	0%	0.90
	CPS ≥ 10	2	0.77	0.61–0.97	0.03	0%	0.37
≥ 2nd-Line	oesophagogastric junction tumor	2	0.71	0.53–0.95	0.02	28%	0.24
combined therapy	Aged < 65 years	2	0.77	0.65–0.92	0.004	0%	0.81
	Male	2	0.85	0.73–1.00	0.04	0%	0.84
	PS 1	2	0.80	0.67–0.96	0.01	0%	0.86
	gastric tumor	2	0.84	0.72–0.99	0.04	0%	0.67
monotherapy	CPS ≥ 10	2	0.67	0.51–0.89	0.005	0%	0.81

### Sensitivity analysis and publication bias

The literature was excluded one by one for sensitivity analyses, revealing that the main outcomes after excluding this research did not differ substantially from past outcomes, indicating low sensitivity and credibility and robustness of the outcomes (Table 4). The outcomes of patient research had an impact on the effect sizes of OS and PFS in this meta-analysis. Nevertheless, overall, by enlarging the sample size for pooled analyses, we have drawn more all-round conclusions about the potency of ICIs in this cohort of individuals with GC/GEJC. These sensitivity analyses did not change the prognostic factors in the entire cohort. Furthermore, owing to the finite number of clinical trials included (n < 10), no obvious publication bias was discovered based on the whole OS and PFS funnel plots (Figures S1), and the OS funnel plot for each subgroup (Figures S2).

**Table 4:** The sensitivity analyses of the studies

Sensitivity analyses	No. of studies	No. of patients	PFS HR, (95% CI)	No. of patients	OS HR, (95% CI)
Total studies	5	2 253	1.17 (0.83–1.64)	2 253	0.91 (0.83–1.00)
JAVELIN Gastric 300 excluded	4	1 882	1.06 (0.72–1.55)	1 882	0.87 (0.79–0.96)
KEYNOTE-061 excluded	4	1 858	1.14 (0.73–1.78)	1 858	0.93 (0.84–1.02)
KEYNOTE-062 (Pem vs Chemo) excluded	4	1 997	1.06 (0.72–1.57)	1 997	0.91 (0.82–1.01)
KEYNOTE-062 (Pem + Chemo vs Chemo) excluded	4	1 996	1.27 (0.88–1.83)	1 996	0.93 (0.84–1.03)
ATTRACTION-4 excluded	4	1 529	1.32 (0.95–1.85)	1 529	0.91 (0.82–1.01)

## Discussion

At present, a large number of retrospective immunotherapy studies have been carried out or are underway to identify new biomarkers. Fehrenbacher et al. showed that atezolizumab was beneficial for survival in patients with high expression of tumor T effector factor and the gene marker IFN-γ [44]. Another study in the US found that patients with a higher proportion of central memory T cells to effector T cells had a longer PFS [45]. Preconditioning lactate dehydrogenase (LDH), C-reactive protein (CRP), neutrophil-to-lymphocyte ratio (NLR), lung immunoprognostic index (according to derived NLR and LDH levels), and the intestinal microbiome may also be potential signatures [46–48]; however, there remains no consensus standard for defining the relevant threshold for some of them. In addition, the potency predictive value of these biomarkers is further proven to be necessary through prospective trials, as they are only found in exploratory or retrospective analyses of small samples. As up-to-date clinical outcomes accumulate, we sought to determine whether additional clinical factors are available and cost-effective to predict the efficacy of immunotherapy in GC/GEJC.

To the best of our knowledge, our meta-analysis is the most accurate and adequate research, and it includes the most recent RCTs, to provide guidance on better identifying patients with GC/GEJC probably benefiting from PD-1/PD-L1 inhibitors.

Previous studies have indicated that ICI-based therapy can prolong the survival of individuals with melanoma or non-small cell lung cancer in any age group [49]. A study also showed that ICIs may be more effective in the elderly, and different mechanisms of action of ICIs in different age groups have been reported [50, 51]. In our meta-analysis, we used 65 years old as the most commonly used age cut-off point to distinguish between older and younger patients and demonstrated that PD-1/PD-L1 inhibitors substantially prolonged survival in patients aged < 65 years compared to chemotherapy. However, PD-1/PD-L1 inhibitors did not substantially improve survival in elderly individuals aged ≥ 65 years. This may be due to the fact that older individuals are closely related the falling of immune system function known as immune-senescence [52], making them unable to regain antitumor activity, and may suffer more frequent or more serious immunotherapy toxicities than younger patients [53, 54]. Therefore, it is necessary to cautiously use anti-PD-1/PD-L1 antibodies, and our current analytical data do not support the large-scale clinical use of ICIs in GC/GEJC patients aged ≥ 65 years.

It is well known that sex differences influence innate and adaptive immune responses [55, 56], possibly reflecting the different potencies of anti-PD-1/PD-L1 antibody therapy in male and female GC/GEJC individuals. Our findings indicate that there may be a correlation between ICIs and sex, with males being more effective. This differential response to immunotherapy has also been observed in male patients with lung cancer [57]. This may be due to the fact that female cancer patients have a stronger immune escape mechanism than male, and therefore more probably develop resistance to immunotherapy [56–58]. Nevertheless, further sub-population analyses showed that a survival benefit was observed with immunotherapy only in the treatment of 1st-line combination, whereas no survival benefits were observed in ≥ 2nd-line or monotherapy. Thus, early combination therapy based on ICIs is recommended for male patients with GC/GEJC. In summary, sex appears to be an appropriate clinical predictor of ICIs. It is worth noting that sex, as a biological variable, should be emphasized in subsequent clinical trials with respect to GC/GEJC of checkpoint inhibitors, and the relationship between sex and efficacy of immunotherapy should no longer be ignored by the scientific community.

Since most RCTs related to PD-1/PD-L1 inhibitors in GC/GEJC exclude individuals with PS ≥ 2, the function of ICIs in these individuals was not analyzed. When we focused on PS 0 and 1, our findings showed that patients with PS 1 seemed to have a longer survival benefit for the treatment of anti-PD-1/anti-PD-L1, with an HR, of 0.93 for patients with PS 0 and 0.89 for patients with PS 1. The preclinical theoretical basis of our findings remains unknown. Results from a previous phase II clinical subgroup analysis of pembrolizumab emphasized that a better PS was related to a higher response rate and longer OS [59]. Mishima et al. also indicated that patients with advanced GC with a PS of 0 had better survival than those with a PS of 1, suggesting that ECOG PS could be used as a predictor of immunotherapy potency [60], which is inconsistent with our results and involves relatively small sample sizes. This may be due to the lack of relevant studies, data on GC/GEJC immunotherapy, and insufficient evidence, which is worthy of close attention in subsequent studies and requires further clarification. Furthermore, patients with PS 1 should be given preference for 1st-line or combination therapy based on ICI as alternative regimens. Therefore, ECOG PS 0 or PS 1 may not be independent predictors of anti-PD-1/PD-L1 antibodies potency.

The Lauren classification distinguishes between two main subtypes of GC: intestinal and diffuse [61]. Diffuse-type GC accounts for approximately 30% of GC cases and is more common in younger individuals [62]. However, there is no systematic review of whether histological type could be meaningful predictors of anti-PD-1/anti-PD-L1 antibodies. We did not find an improvement in survival after ICI therapy in patients with intestinal or diffuse types. In addition, our results showed that individuals did not benefit from anti-PD-1/PD-L1 antibody therapy regardless of previous gastrectomy status or primary tumor sites. Nevertheless, Dong et al. found that individuals who had previously undergone gastrectomy had a relatively better prognosis than those who had not. They thought gastrectomy leads to an imbalance in Th17/Treg and increased PD-1/PD-L1 expression. Blocking the PD-1 and PD-L1 pathways can alleviate Th17/Treg cell imbalance resulting from gastric surgery [63]. Therefore, the following researches may pay more attention to whether gastrectomy affects the efficacy of immunotherapy. For GC patients treated with anti-PD-1/PD-L1 antibodies, early combination treatment based on ICI is recommended. Furthermore, our analysis suggested that GEJC patients obtained OS improvement in ≥ 2nd-line therapy based on ICI, which further illustrated the significance of follow-up treatment.

The expression of PD-L1 has been investigated and demonstrated to be a moderately effective predictor of ICIs potency in GC patients, but its prognostic significance is not clear. A review of 15 published studies showed that in 11 studies, the expression of PD-L1 on the combined positive score (CPS) was a negative prognostic factor for OS, and in three studies, it was a positive prognostic factor, one of which reported no prognostic significance [64]. In our analysis, the potency of the anti-PD-1/anti-PD-L1 antibody on OS in the subgroup with a higher CPS cut-off was better than that in the subgroup with a lower CPS cut-off. Further, the benefits of ICIs based 1st-line treatment and monotherapy on OS were observed in subpopulations with a CPS ≥ 10. In addition, our analysis results revealed that in terms of the tumor proportion score (TPS), that is, the percentage of positive tumor cells only, negative PD-L1 expression (TPS < 1%), or positive PD-L1 expression (TPS ≥ 1%) showed no improvement in OS after application of anti-PD-1/anti-PD-L1 antibodies. This may be due to the fact that a small number of patients were analyzed, or to inherent limitations of the trials themselves, such as the confounding factors and systematic bias risk, as well as the performance differences between different PD-L1 measurement methods [65], all caused the result of which show that PD-L1 TPS does not appear to be a suitable biomarker for predicting ICIs. Kulangara et al. assessed the capacity of PD-L1 CPS and TPS as biomarkers to predict pembrolizumab potency in the KEYNOTE-059 trial, estimating pembrolizumab monotherapy and combination therapy in patients with recurrent or metastatic GC/GEJC [66]. The study revealed that the objective response to pembrolizumab was substantially correlated with the CPS, according to the PD-L1 CPS truncation value of 1, but not with the TPS. Although the clinical significance of CPS and the best cut-off point for ICI treatment have not been determined due to the rapid clinical development of ICIs and based on limited data, these observations support ICIs as another treatment option for GC/GEJC in PD-L1 CPS ≥ 1 individuals [67] according to the immunohistochemistry of PD-L1 [68]. Compared with TPS, PD-L1 CPS is deemed as a highly reliable and reproducible scoring method to predict the response to PD-1/PD-L1 inhibitors in GC/GEJC individuals [66, 69]. Currently, CPS combines PD-L1 expression in tumor and immune cells into a single score, and pathologists are able to evaluate them directly without having to score them [70]. This allows CPS to play a role in GC, where the expression of PD-L1 is driven by immune cells, tumor cells, or both. In addition, CPS has nothing to do with organizational structure [71], and it is hoped that CPS will play a role in cytology or digestion of tumor samples. Therefore, we expect that CPS could be an effective diagnostic tool in the field of GC/GEJC immunotherapy.

In our meta-analysis, line of treatment also predicted the clinical results of ICIs. We found that when the number of previous system schemes was 0, the survival benefits of immunotherapy were achieved; however, in this study, two related studies in GC and GEJC on the treatment of ≥ 2nd-line, involving JAVELIN Gastric 300 [40] and KEYNOTE-061 [41], showed that OS did not improve with the patient’s HR, of 0.96. This may be due to latent immunosuppression and severe adverse reactions caused by conventional chemotherapy or previously applied targeted therapy, which is likely to have a negative effect on subsequent immunotherapy [72]. For GC/GEJC patients, OS may be affected by the previous treatment route rather than the efficacy of the experimental drugs themselves. Therefore, generally speaking, the treatment of anti-PD-1/anti-PD-L1 is not satisfactory in the ≥ 2nd-line application of GC and GEJC.

While our paper has produced promising insights, we must admit that the limitations of the present study are evident in many aspects. First, the limitation of this paper is that the number of trials that meet the analysis conditions is limited. However, the sample size was > 2000, so it was comparable in all trials. And the sample size of the JAVELIN 300 was the smallest (*n* = 371), indicating that there was no obvious publication bias or asymmetry. Second, we collected the trial outcomes from the published papers but could not obtain the raw data of each patient, possibly causing a bias in data analysis. Under the premise of more relevant research, extensive clinical studies and synthesis analyses of larger samples can be developed to further confirm the potency of ICIs therapy in GC/GEJC. Third, this meta-analysis was based on usable survival HR, assuming the proportion of hazards. In particular, in the KEYNOTE-061 and 062 trials, there was an obvious crossover between the two survival curves after 6–8 months, which makes the HR, measurement unreliable, and thus the combined HR, of the meta-analysis unreliable [73, 74]. The intersection of the survival curves occurred in immunotherapy trials, indicating that there are two different patient subgroups, and the response to ICI is the opposite [75]. Fourth, the limitations of further research were probably attributable to the lack of subgroup data on a few PD-1/PD-L1 inhibitors. Differences in efficacy among subgroups such as microsatellite instability (MSI) status, smoking status, and alcohol use may be the next direction for continued research. Data from the implementation of future trials may reinforce these observations. Fifth, the weakness and complexity of the results of this study lies in the probable differences in pharmacology between anti-PD-1 and anti-PD-L1 antibodies, and the different antibodies for evaluating PD-L1 status used in the RCTs. Finally, in view of the subgroup analysis, the findings should be interpreted with caution because of the small number of individuals analyzed. Hence, further validation studies are essential in GC/GEJC patients treated with PD-1/PD-L1 inhibitors.

In summary, PD-1/PD-L1 inhibitors improve OS but not PFS compared with chemotherapy in GC/GEJC. Age and sex could be used to predict the treatment potency of PD-1/PD-L1 inhibitors in GC/GEJC. With more phase III trials published in the future, the subgroup analyses may provide more accurate and comprehensive subgroup information to help us select the appropriate treatment population.

## Supplementary Information

**Additional file 1: Figure S1.** Funnel plots for (A) OS and (B) PFS between anti-PD-1/PD-L1 therapy and chemotherapy. **Figure S2. **Funnel plots for OS in the subgroup with respect to (A) age group, (B) gender, (C) PS score, (D) Lauren histological type, (E) previous gastrectomy status, (F) primary tumour sites, (G) PD-L1 TPS, (H) PD-L1 CPS, (I) treatment line.

## Data Availability

The original contributions of the study are included in the article/supplementary material. Further inquiries can be directed to the corresponding authors.

## References

[1] Bray F, Ferlay J, Soerjomataram I, Siegel RL, Torre LA, Jemal A (2018). Global cancer statistics 2018: GLOBOCAN estimates of incidence and mortality worldwide for 36 cancers in 185 countries. CA Cancer J Clin.

[2] Thrift AP, El-Serag HB (2020). Burden of Gastric Cancer. Clin Gastroenterol Hepatol.

[3] Fuchs CS, Tomasek J, Yong CJ, Dumitru F, Passalacqua R, Goswami C (2014). Ramucirumab monotherapy for previously treated advanced gastric or gastro-oesophageal junction adenocarcinoma (REGARD): an international, randomised, multicentre, placebo-controlled, phase 3 trial. Lancet.

[4] Wilke H, Muro K, Van Cutsem E, Oh SC, Bodoky G, Shimada Y (2014). Ramucirumab plus paclitaxel versus placebo plus paclitaxel in patients with previously treated advanced gastric or gastro-oesophageal junction adenocarcinoma (RAINBOW): a double-blind, randomised phase 3 trial. Lancet Oncol.

[5] Van Laethem JL, Carneiro F, Ducreux M, Messman H, Lordick F, Ilson DH (2016). The multidisciplinary management of gastro-oesophageal junction tumours: European Society of Digestive Oncology (ESDO): Expert discussion and report from the 16th ESMO World Congress on Gastrointestinal Cancer. Barcelona Dig Liver Dis.

[6] Fuchs CS, Muro K, Tomasek J, Van Cutsem E, Cho JY, Oh SC (2017). Prognostic Factor Analysis of Overall Survival in Gastric Cancer from Two Phase III Studies of Second-line Ramucirumab (REGARD and RAINBOW) Using Pooled Patient Data. J Gastric Cancer.

[7] Pavel M, Öberg K, Falconi M, Krenning EP, Sundin A, Perren A (2020). Gastroenteropancreatic neuroendocrine neoplasms: ESMO Clinical Practice Guidelines for diagnosis, treatment and follow-up. Ann Oncol.

[8] Dittmar Y, Settmacher U (2015). Individualized treatment of gastric cancer: Impact of molecular biology and pathohistological features. World J Gastrointest Oncol.

[9] Al-Batran SE, Hartmann JT, Probst S, Schmalenberg H, Hollerbach S, Hofheinz R (2008). Phase III trial in metastatic gastroesophageal adenocarcinoma with fluorouracil, leucovorin plus either oxaliplatin or cisplatin: a study of the Arbeitsgemeinschaft Internistische Onkologie. J Clin Oncol.

[10] Bang YJ, Van Cutsem E, Feyereislova A, Chung HC, Shen L, Sawaki A (2010). Trastuzumab in combination with chemotherapy versus chemotherapy alone for treatment of HER2-positive advanced gastric or gastro-oesophageal junction cancer (ToGA): a phase 3, open-label, randomised controlled trial. Lancet.

[11] Kang JH, Lee SI, Lim DH, Park KW, Oh SY, Kwon HC (2012). Salvage chemotherapy for pretreated gastric cancer: a randomized phase III trial comparing chemotherapy plus best supportive care with best supportive care alone. J Clin Oncol.

[12] Ford HE, Marshall A, Bridgewater JA, Janowitz T, Coxon FY, Wadsley J (2014). Docetaxel versus active symptom control for refractory oesophagogastric adenocarcinoma (COUGAR-02): an open-label, phase 3 randomised controlled trial. Lancet Oncol.

[13] Wagner AD, Syn NL, Moehler M, Grothe W, Yong WP, Tai BC (2017). Chemotherapy for advanced gastric cancer. Cochrane Database Syst Rev.

[14] Pardoll DM (2012). The blockade of immune checkpoints in cancer immunotherapy. Nat Rev Cancer.

[15] Sharma P, Allison JP (2015). Immune checkpoint targeting in cancer therapy: toward combination strategies with curative potential. Cell.

[16] Sun C, Mezzadra R, Schumacher TN (2018). Regulation and Function of the PD-L1 Checkpoint. Immunity.

[17] Tian Y, Zhai X, Han A, Zhu H, Yu J (2019). Potential immune escape mechanisms underlying the distinct clinical outcome of immune checkpoint blockades in small cell lung cancer. J Hematol Oncol.

[18] Page DB, Postow MA, Callahan MK, Allison JP, Wolchok JD (2014). Immune modulation in cancer with antibodies. Annu Rev Med.

[19] Brahmer J, Reckamp KL, Baas P, Crinò L, Eberhardt WE, Poddubskaya E (2015). Nivolumab versus Docetaxel in Advanced Squamous-Cell Non-Small-Cell Lung Cancer. N Engl J Med.

[20] Motzer RJ, Escudier B, McDermott DF, George S, Hammers HJ, Srinivas S (2015). Nivolumab versus Everolimus in Advanced Renal-Cell Carcinoma. N Engl J Med.

[21] Weber J, Mandala M, Del Vecchio M, Gogas HJ, Arance AM, Cowey CL (2017). Adjuvant Nivolumab versus Ipilimumab in Resected Stage III or IV Melanoma. N Engl J Med.

[22] Ni X, Xing Y, Sun X, Suo J (2020). The safety and efficacy of anti-PD-1/anti-PD-L1 antibody therapy in the treatment of previously treated, advanced gastric or gastro-oesophageal junction cancer: A meta-analysis of prospective clinical trials. Clin Res Hepatol Gastroenterol.

[23] Hegde PS, Chen DS (2020). Top 10 Challenges in Cancer Immunotherapy. Immunity.

[24] Böger C, Behrens HM, Mathiak M, Krüger S, Kalthoff H, Röcken C (2016). PD-L1 is an independent prognostic predictor in gastric cancer of Western patients. Oncotarget.

[25] Derks S, Liao X, Chiaravalli AM, Xu X, Camargo MC, Solcia E (2016). Abundant PD-L1 expression in Epstein-Barr Virus-infected gastric cancers. Oncotarget.

[26] West H, McCleod M, Hussein M, Morabito A, Rittmeyer A, Conter HJ (2019). Atezolizumab in combination with carboplatin plus nab-paclitaxel chemotherapy compared with chemotherapy alone as first-line treatment for metastatic non-squamous non-small-cell lung cancer (IMpower130): a multicentre, randomised, open-label, phase 3 trial. Lancet Oncol.

[27] Chen TT. Milestone survival: a potential intermediate endpoint for immune checkpoint inhibitors. J Natl Cancer Inst. 2015;107(9). 10.1093/jnci/djv156. 10.1093/jnci/djv156 PMC4836810 26113579

[28] Rittmeyer A, Gandara D, Kowanetz M, Mok T, Shames DS (2018). Blood-Based Biomarkers for Cancer Immunotherapy: Tumor Mutational Burden in Blood (bTMB) is Associated with Improved Atezolizumab (atezo) Efficacy in 2L+ NSCLC (POPLAR and OAK). Pneumologie.

[29] Lantuejoul S, Sound-Tsao M, Cooper WA, Girard N, Hirsch FR, Roden AC (2020). PD-L1 Testing for Lung Cancer in 2019: Perspective From the IASLC Pathology Committee. J Thorac Oncol.

[30] Munari E, Mariotti FR, Quatrini L, Bertoglio P, Tumino N, Vacca P, et al. PD-1/PD-L1 in cancer: pathophysiological, diagnostic and therapeutic aspects. Int J Mol Sci. 2021;22(10). 10.3390/ijms22105123. 10.3390/ijms22105123 PMC8151504 34066087

[31] Licitra L, Locati LD, Bossi P (2008). Optimizing approaches to head and neck cancer. Metastatic head and neck cancer: new options. Ann Oncol.

[32] Janjigian YY, Sanchez-Vega F, Jonsson P, Chatila WK, Hechtman JF, Ku GY (2018). Genetic predictors of response to systemic therapy in Esophagogastric Cancer. Cancer Discov.

[33] Chen C, Zhang F, Zhou N, Gu YM, Zhang YT, He YD (2019). Efficacy and safety of immune checkpoint inhibitors in advanced gastric or gastroesophageal junction cancer: a systematic review and meta-analysis. Oncoimmunology.

[34] Liu C, Wang W, Yang J, Song P, Li F, Liu B (2020). The efficacy and safety comparison of PD-1/PD-L1 antibody, chemotherapy and supportive treatment for pretreated advanced esophagogastric cancer: a network meta-analysis. Ann Palliat Med.

[35] Chen K, Wang X, Yang L, Chen Z (2021). The Anti-PD-1/PD-L1 immunotherapy for gastric esophageal cancer: a systematic review and meta-analysis and literature review. Cancer Control.

[36] Zhao Q, Yu J, Meng X (2019). A good start of immunotherapy in esophageal cancer. Cancer Med.

[37] Lu Y, Guan L, Xu M, Wang F (2021). The efficacy and safety of antibodies targeting PD-1 for treatment in advanced esophageal cancer: A systematic review and meta-analysis. Transl Oncol.

[38] Janjigian YY, Shitara K, Moehler M, Garrido M, Salman P, Shen L (2021). First-line nivolumab plus chemotherapy versus chemotherapy alone for advanced gastric, gastro-oesophageal junction, and oesophageal adenocarcinoma (CheckMate 649): a randomised, open-label, phase 3 trial. Lancet.

[39] Liberati A, Altman DG, Tetzlaff J, Mulrow C, Gøtzsche PC, Ioannidis JP (2009). The PRISMA statement for reporting systematic reviews and meta-analyses of studies that evaluate healthcare interventions: explanation and elaboration. BMJ.

[40] Bang YJ, Ruiz EY, Van Cutsem E, Lee KW, Wyrwicz L, Schenker M (2018). Phase III, randomised trial of avelumab versus physician's choice of chemotherapy as third-line treatment of patients with advanced gastric or gastro-oesophageal junction cancer: primary analysis of JAVELIN Gastric 300. Ann Oncol.

[41] Shitara K, Özgüroğlu M, Bang YJ, Di Bartolomeo M, Mandalà M, Ryu MH (2018). Pembrolizumab versus paclitaxel for previously treated, advanced gastric or gastro-oesophageal junction cancer (KEYNOTE-061): a randomised, open-label, controlled, phase 3 trial. Lancet.

[42] Shitara K, Van Cutsem E, Bang YJ, Fuchs C, Wyrwicz L, Lee KW (2020). Efficacy and Safety of Pembrolizumab or Pembrolizumab Plus Chemotherapy vs Chemotherapy Alone for Patients With First-line, Advanced Gastric Cancer: The KEYNOTE-062 Phase 3 Randomized Clinical Trial. JAMA Oncol.

[43] Kang YK, Chen LT, Ryu MH, Oh DY, Oh SC, Chung HC (2022). Nivolumab plus chemotherapy versus placebo plus chemotherapy in patients with HER2-negative, untreated, unresectable advanced or recurrent gastric or gastro-oesophageal junction cancer (ATTRACTION-4): a randomised, multicentre, double-blind, placebo-controlled, phase 3 trial. Lancet Oncol.

[44] Fehrenbacher L, Spira A, Ballinger M, Kowanetz M, Vansteenkiste J, Mazieres J (2016). Atezolizumab versus docetaxel for patients with previously treated non-small-cell lung cancer (POPLAR): a multicentre, open-label, phase 2 randomised controlled trial. Lancet.

[45] Manjarrez-Orduño N, Menard LC, Kansal S, Fischer P, Kakrecha B, Jiang C (2018). Circulating T Cell Subpopulations Correlate With Immune Responses at the Tumor Site and Clinical Response to PD1 Inhibition in Non-Small Cell Lung Cancer. Front Immunol.

[46] Donskov F (2013). Immunomonitoring and prognostic relevance of neutrophils in clinical trials. Semin Cancer Biol.

[47] Mezquita L, Auclin E, Ferrara R, Charrier M, Remon J, Planchard D (2018). Association of the lung immune prognostic index with immune checkpoint inhibitor outcomes in patients with advanced non-small cell lung cancer. JAMA Oncol.

[48] Iivanainen S, Ahvonen J, Knuuttila A, Tiainen S, Koivunen JP (2019). Elevated CRP levels indicate poor progression-free and overall survival on cancer patients treated with PD-1 inhibitors. ESMO Open.

[49] Elias R, Giobbie-Hurder A, McCleary NJ, Ott P, Hodi FS, Rahma O (2018). Efficacy of PD-1 & PD-L1 inhibitors in older adults: a meta-analysis. J Immunother Cancer.

[50] Kugel CH, Douglass SM, Webster MR, Kaur A, Liu Q, Yin X (2018). Age Correlates with Response to Anti-PD1, Reflecting Age-Related Differences in Intratumoral Effector and Regulatory T-Cell Populations. Clin Cancer Res.

[51] Wu Q, Wang Q, Tang X, Xu R, Zhang L, Chen X (2019). Correlation between patients' age and cancer immunotherapy efficacy. Oncoimmunology.

[52] Elias R, Karantanos T, Sira E, Hartshorn KL (2017). Immunotherapy comes of age: Immune aging & checkpoint inhibitors. J Geriatr Oncol.

[53] Jagger A, Shimojima Y, Goronzy JJ, Weyand CM (2014). Regulatory T cells and the immune aging process: a mini-review. Gerontology.

[54] Bhandari S, Gill AS, Perez CA, Jain D (2018). Management of immunotherapy toxicities in older adults. Semin Oncol.

[55] Oertelt-Prigione S (2012). The influence of sex and gender on the immune response. Autoimmun Rev.

[56] Klein SL, Flanagan KL (2016). Sex differences in immune responses. Nat Rev Immunol.

[57] Conforti F, Pala L, Bagnardi V, De Pas T, Martinetti M, Viale G (2018). Cancer immunotherapy efficacy and patients' sex: a systematic review and meta-analysis. Lancet Oncol.

[58] Schreiber RD, Old LJ, Smyth MJ (2011). Cancer immunoediting: integrating immunity's roles in cancer suppression and promotion. Science.

[59] Le DT, Uram JN, Wang H, Bartlett BR, Kemberling H, Eyring AD (2015). PD-1 Blockade in tumors with mismatch-repair deficiency. N Engl J Med.

[60] Mishima S, Kawazoe A, Nakamura Y, Sasaki A, Kotani D, Kuboki Y (2019). Clinicopathological and molecular features of responders to nivolumab for patients with advanced gastric cancer. J Immunother Cancer.

[61] Lauren P (1965). The two histological main types of gastric carcinoma: diffuse and so-called intestinal-type carcinoma. An attempt at a histo-clinical classification. Acta Pathol Microbiol Scand.

[62] Lordick F, Janjigian YY (2016). Clinical impact of tumour biology in the management of gastroesophageal cancer. Nat Rev Clin Oncol.

[63] Dong L, Zheng X, Wang K, Wang G, Zou H (2018). Programmed death 1/programmed cell death-ligand 1 pathway participates in gastric surgery-induced imbalance of T-helper 17/regulatory T cells in mice. J Trauma Acute Care Surg.

[64] Gu L, Chen M, Guo D, Zhu H, Zhang W, Pan J (2017). PD-L1 and gastric cancer prognosis: A systematic review and meta-analysis. PLoS One.

[65] Aguiar PN, De Mello RA, Hall P, Tadokoro H, Lima LG (2017). PD-L1 expression as a predictive biomarker in advanced non-small-cell lung cancer: updated survival data. Immunotherapy.

[66] Kulangara K, Zhang N, Corigliano E, Guerrero L, Waldroup S, Jaiswal D (2019). Clinical Utility of the Combined Positive Score for Programmed Death Ligand-1 Expression and the Approval of Pembrolizumab for Treatment of Gastric Cancer. Arch Pathol Lab Med.

[67] Brar G, Shah MA (2019). The role of pembrolizumab in the treatment of PD-L1 expressing gastric and gastroesophageal junction adenocarcinoma. Therap Adv Gastroenterol.

[68] Ju X, Zhang H, Zhou Z, Wang Q (2020). Regulation of PD-L1 expression in cancer and clinical implications in immunotherapy. Am J Cancer Res.

[69] Park Y, Koh J, Na HY, Kwak Y, Lee KW, Ahn SH (2020). PD-L1 Testing in Gastric Cancer by the Combined Positive Score of the 22C3 PharmDx and SP263 Assay with Clinically Relevant Cut-offs. Cancer Res Treat.

[70] Driver BR, Miller RA, Miller T, Deavers M, Gorman B, Mody D (2017). Programmed Death Ligand-1 (PD-L1) Expression in Either Tumor Cells or Tumor-Infiltrating Immune Cells Correlates With Solid and High-Grade Lung Adenocarcinomas. Arch Pathol Lab Med.

[71] Yuan P, Guo C, Li L, Guo L, Zhang F, Ying J (2021). The Reproducibility of Histopathologic Assessments of Programmed Cell Death-Ligand 1 Using Companion Diagnostics in NSCLC. JTO Clin Res Rep.

[72] Reck M, Remon J, Hellmann MD (2022). First-Line Immunotherapy for Non-Small-Cell Lung Cancer. J Clin Oncol.

[73] Tomeczkowski J, Lange A, Güntert A, Thilakarathne P, Diels J, Xiu L (2015). Converging or Crossing Curves: Untie the Gordian Knot or Cut it? Appropriate Statistics for Non-Proportional Hazards in Decitabine DACO-016 Study (AML). Adv Ther.

[74] Uno H, Claggett B, Tian L, Inoue E, Gallo P, Miyata T (2014). Moving beyond the hazard ratio in quantifying the between-group difference in survival analysis. J Clin Oncol.

[75] Bellmunt J, de Wit R, Vaughn DJ, Fradet Y, Lee JL, Fong L (2017). Pembrolizumab as second-line therapy for advanced urothelial carcinoma. N Engl J Med.

