# Supplementary material for: Efficacy of PD-1/PD-L1 inhibitors in gastric or gastro-oesophageal junction cancer based on clinical characteristics: a meta-analysis
Source: BMC Cancer. 2023 Feb 10;23:143. doi: 10.1186/s12885-023-10605-y (PMC9921519; doi:10.1186/s12885-023-10605-y)
Supplement: Supplementary file 1 — Additional file 1: Figure S1. Funnel plots for (A) OS and (B) PFS between anti-PD-1/PD-L1 therapy and chemotherapy. Figure S2. Funnel plots for OS in the subgroup with respect to (A) age group, (B) gender, (C) PS score, (D) Lauren histological type, (E) previous gastrectomy status, (F) primary tumour sites, (G) PD-L1 TPS, (H) PD-L1 CPS, (I) treatment line. [file 12885_2023_10605_MOESM1_ESM.pdf]

A

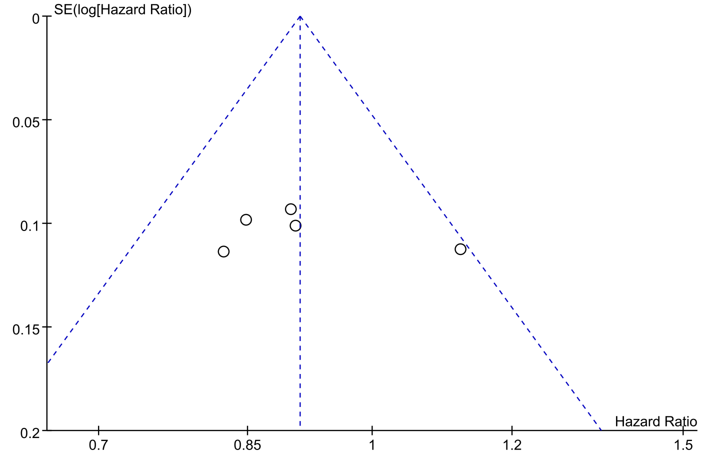

B

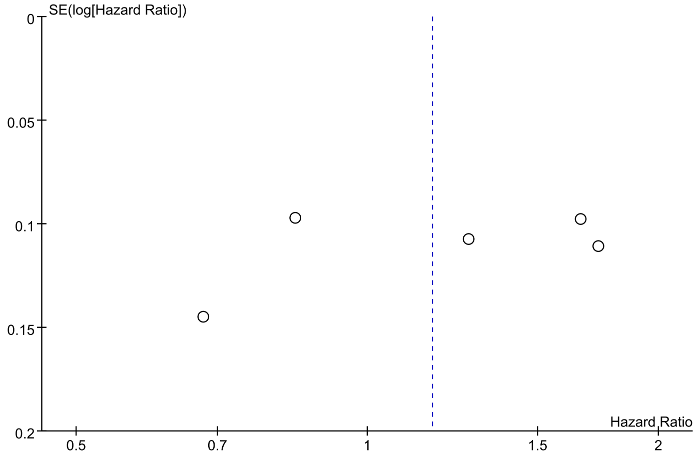

Figure S1: Funnel plots for (A) OS and (B) PFS between anti-PD-1/PD-L1 therapy and chemotherapy.

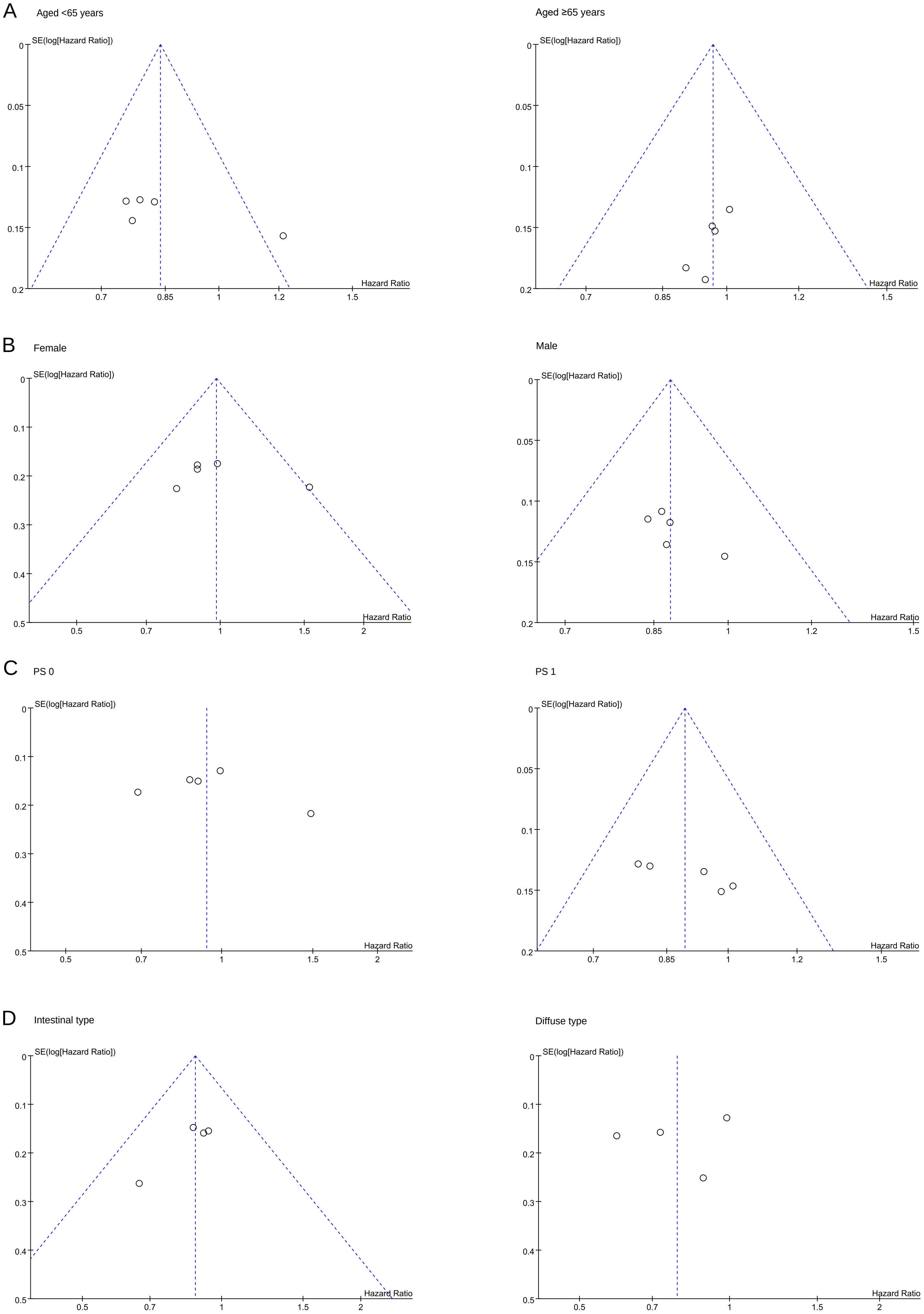
A
Aged <65 years
Aged ≥65 years
B
Female
Male
C
PS 0
PS 1
D
Intestinal type
Diffuse type
SE(log[Hazard Ratio])
Hazard Ratio

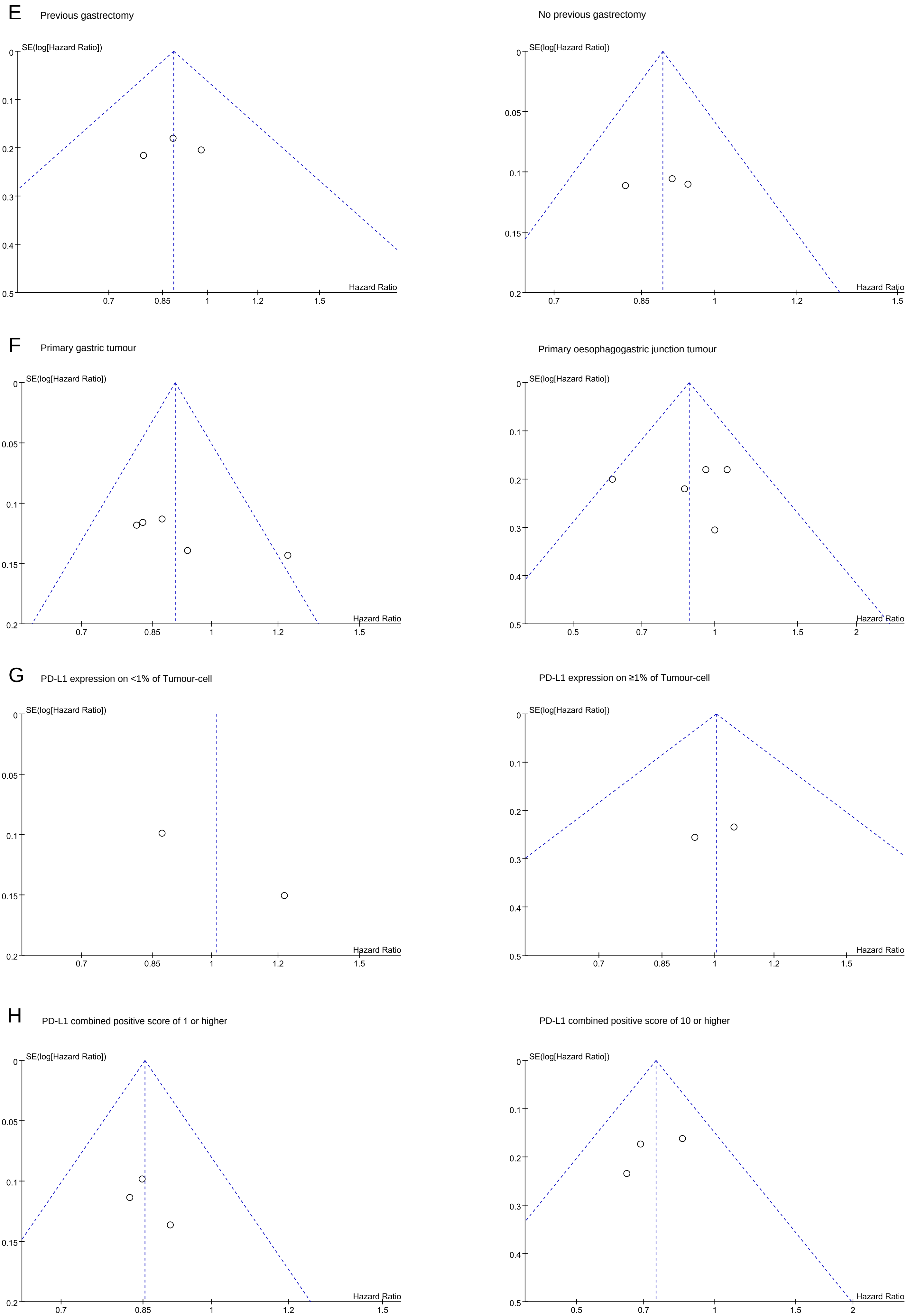

E
Previous gastrectomy
No previous gastrectomy
F
Primary gastric tumour
Primary oesophagogastric junction tumour
G
PD-L1 expression on <1% of Tumour-cell
PD-L1 expression on ≥1% of Tumour-cell
H
PD-L1 combined positive score of 1 or higher
PD-L1 combined positive score of 10 or higher
SE(log[Hazard Ratio])
Hazard Ratio

I

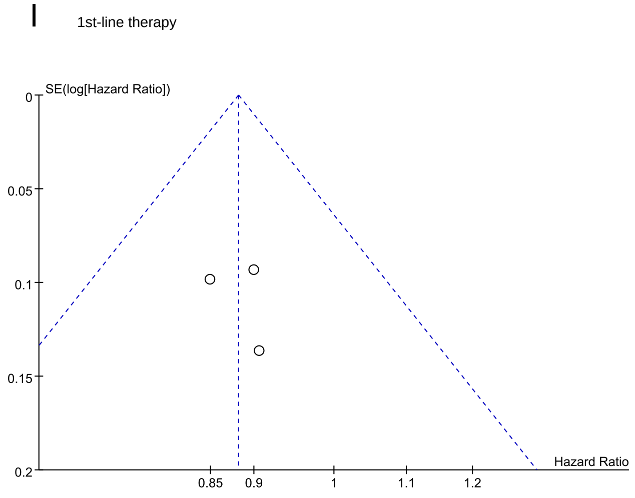

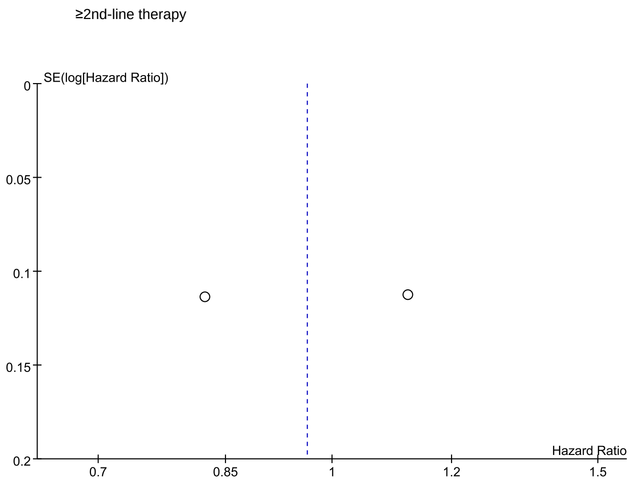

Figure S2: Funnel plots for OS in the subgroup with respect to (A) age group, (B) gender, (C) PS score, (D) Lauren histological type, (E) previous gastrectomy status, (F) primary tumour sites, (G) PD-L1 TPS, (H) PD-L1 CPS, (I) treatment line.
